# Supplementary figures and images for: Intubation with channeled versus non-channeled video laryngoscopes in simulated difficult airway by junior doctors in an out-of-hospital setting: A crossover manikin study
Source: PLoS One. 2019 Oct 22;14(10):e0224017. doi: 10.1371/journal.pone.0224017 (PMC6805049; doi:10.1371/journal.pone.0224017)

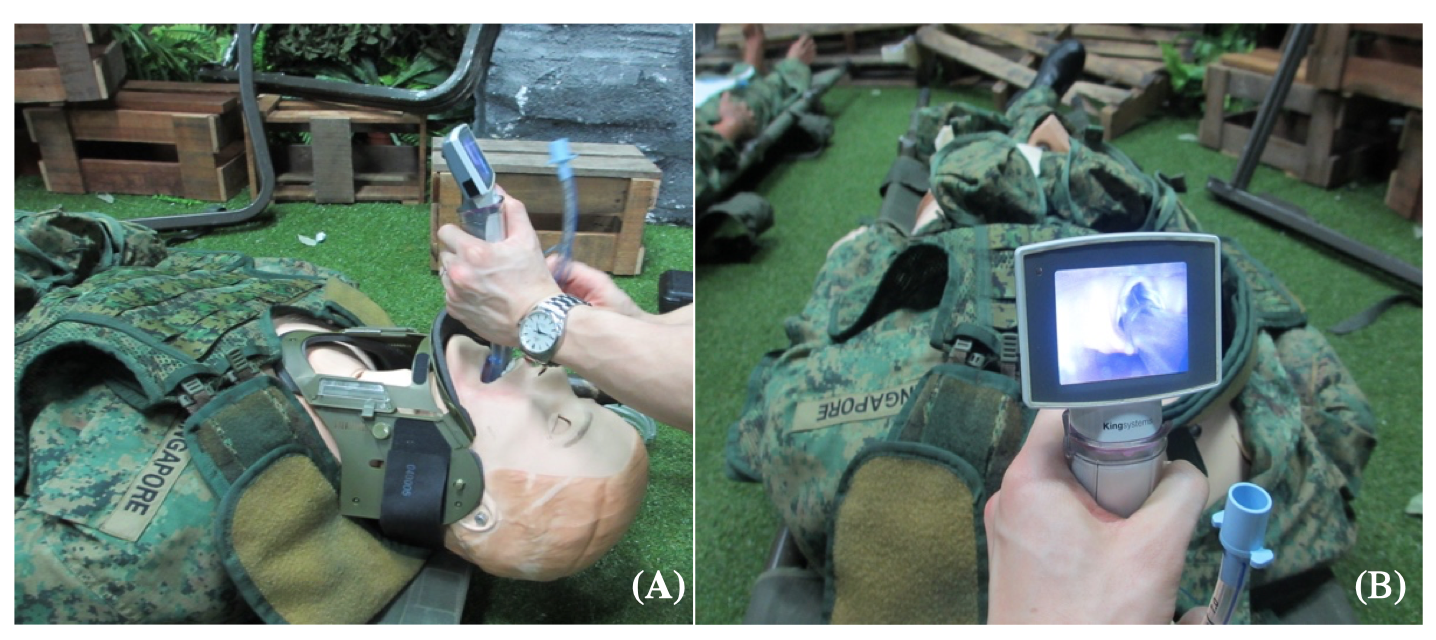

Supplement: S1 Fig — (PNG) [file pone.0224017.s001.png]
