# Supplementary material for: Intubation with channeled versus non-channeled video laryngoscopes in simulated difficult airway by junior doctors in an out-of-hospital setting: A crossover manikin study
Source: PLoS One. 2019 Oct 22;14(10):e0224017. doi: 10.1371/journal.pone.0224017 (PMC6805049; doi:10.1371/journal.pone.0224017)
Supplement: S1 Table — (DOCX) [file pone.0224017.s003.docx]

**Table 1. Intubation sequence based on a 3-period, 3-treatment crossover design.**

| Sequence | Period 1 | Period 2 | Period 3 |
| --- | --- | --- | --- |
| A | McGrath | King Vision channeled | King Vision  non-channeled |
| B | McGrath | King Vision  non-channeled | King Vision channeled |
| C | King Vision channeled | King Vision  non-channeled | McGrath |
| D | King Vision channeled | McGrath | King Vision  non-channeled |
| E | King Vision  non-channeled | King Vision channeled | McGrath |
| F | King Vision  non-channeled | McGrath | King Vision channeled |
