# Supplementary material for: Intubation with channeled versus non-channeled video laryngoscopes in simulated difficult airway by junior doctors in an out-of-hospital setting: A crossover manikin study
Source: PLoS One. 2019 Oct 22;14(10):e0224017. doi: 10.1371/journal.pone.0224017 (PMC6805049; doi:10.1371/journal.pone.0224017)
Supplement: S2 Table — (DOCX) [file pone.0224017.s004.docx]

**Table 2. Comparison of successful intubation and time-to-visualization between the King Vision channeled and non-channeled groups, and McGrath.** Values are number (proportion) or mean (standard deviation).

|  | King Vision channeled (n=105) | King Vision non-channeled  (n=105) | McGrath (n=105) | p-value |
| --- | --- | --- | --- | --- |
| Successful intubation | 90 (85.7%) | 26 (24.8%) | 87 (82.9%) | <0.001^i^ |
| Time to visualization (seconds) | 12.1 (±7.3) | 10.3 (±12.9) | 13.9 (±11.7) | 0.054^ii^ |

^i^  p <0.001 for King Vision channeled versus non-channeled, and McGrath versus King Vision non-channeled; p=0.569 for King Vision channeled versus McGrath

^ii^ p =0.651 for King Vision channeled versus non-channeled, p =0.047 McGrath versus King Vision non-channeled; p=0.704 for King Vision channeled versus McGrath
