# Supplementary material for: Intubation with channeled versus non-channeled video laryngoscopes in simulated difficult airway by junior doctors in an out-of-hospital setting: A crossover manikin study
Source: PLoS One. 2019 Oct 22;14(10):e0224017. doi: 10.1371/journal.pone.0224017 (PMC6805049; doi:10.1371/journal.pone.0224017)
Supplement: S3 Table — (DOCX) [file pone.0224017.s005.docx]

**Table 3.** **Comparison of time-to-intubation for King Vision channeled and non-channeled groups, and McGrath.** Results are expressed as mean (standard deviation).

|  | King Vision channeled  (n=90) | King Vision  non-channeled  (n=26) | McGrath  (n=87) | p-value |
| --- | --- | --- | --- | --- |
| Time to intubation (seconds) | 41.3 (±20.2) | 53.8 (±23.8) | 38.5 (±18.7) | 0.004^i^ |

^i^ p=0.017 for King Vision channeled versus King Vision non-channeled; p=0.002 for McGrath versus King Vision non-channeled; p=1.000 for King Vision channeled versus McGrath
